# Neighbourhood greenspace is associated with a slower decline in physical activity in older adults: A prospective cohort study

**DOI:** 10.1016/j.ssmph.2016.09.006

**Published:** 2016-09-14

**Authors:** Alice M. Dalton, Nick Wareham, Simon Griffin, Andrew P. Jones

**Affiliations:** a Norwich Medical School, University of East Anglia, Norwich Research Park, Norwich NR4 7TJ, UK; b Medical Research Council Epidemiology Unit, University of Cambridge School of Clinical Medicine, Box 285 Institute of Metabolic Science, Cambridge Biomedical Campus, Cambridge CB2 0QQ, UK

**Keywords:** Physical activity, Greenspace exposure, Older adults, Dog walking

## Abstract

Maintaining physical activity in later life is important for maintaining health and function. Activity outdoors, such as walking, jogging and cycling, may provide an accessible, sociable and practical solution, but maintaining outdoor mobility may be a challenge in later life. Providing green environments which are supportive of physical activity may facilitate this, yet research into how greenspace could be best used is inconclusive. This study evaluates the role of greenspace in protecting against decline in physical activity over time in older adults.

Data from the European Prospective Investigation of Cancer Norfolk, UK, cohort 1993–2009 (N=15,672) was used. Linear regression modelling was used to examine the association between exposure to greenspace in the home neighbourhood and change in overall, recreational and outdoor physical activity measured in terms of metabolic equivalent cost (MET) in hours/week. Mediation analysis was conducted to assess if dog walking explained the relationship between greenspace and physical activity change. Models were adjusted for known and hypothesised confounders.

People living in greener neighbourhoods experienced less of a decline in physical activity than those living in less green areas. Comparing change for those living in the greenest versus least green quartiles, participants showed a difference in overall physical activity of 4.21 MET hours/week (trend P=0.001), adjusted for baseline physical activity, age, sex, BMI, social class and marital status. This difference was 4.03 MET hours/week for recreational physical activity (trend P<0.001) and 1.28 MET hours/week for outdoor physical activity (trend P=0.007). Dog walking partially mediated the association between greenspace and physical activity change, by 22.6% for overall, 28.1% for recreational and 50.0% for outdoor physical activity (all P<0.001).

Greenspace in the home neighbourhood may be protective against decline in physical activity among older people as they age. Dog walking is a potential mechanism in this relationship, and warrants further investigation as a way of maintaining physical activity in later life.

## Highlights

• Change in physical activity was examined for 15,672 people over a 7.5-year period.
• Domains of overall, recreational and outdoor physical activity were quantified.
• Neighbourhood greenspace may protect against physical activity decline in older age.
• Dog walking is a potential mechanism in this relationship and should be investigated.

## 1 Introduction

Retaining physical and psychological function in later life is an important part of ‘active ageing’ (World Health Organisation, 2002) through the ability to maintain independence in activities of daily living (McCusker, Kakuma, & Abrahamowicz, 2002). Remaining physically active helps prevent the age-related decline in physical (Paterson & Warburton, 2010) and cognitive (Carvalho et al., 2014, Blondell et al., 2014) function, and associated loss of independence (Paterson, Govindasamy, Vidmar, Cunningham, & Koval, 2004). However, we become less physically active as we age, particularly during the transition into retirement where increased leisure time activity typically does not compensate the loss of work-based activity (Zantinge, van den Berg, Smit, & Picavet, 2014). Early intervention is necessary to encourage physical activity before the process of functional decline begins (Hebert, 1997).

Outdoor recreation, including walking, jogging and cycling, may be the best source of physical activity for older people, as it can be incorporated in daily life (Ogilvie et al., 2007), has been shown to lead to a decrease in all-cause mortality and chronic disease (Zhao et al., 2015), it facilitates social contact (World Health Organisation, 2002), can result in higher levels of physical activity (Kerr et al., 2012) and may provide additional health benefits over engaging in activity indoors (Thompson Coon et al., 2011). However, maintaining outdoor mobility may be a challenge in later life, as individuals are at increased risk of sensory or physical impairment with age, and may be subject to environmental barriers (Mollenkopf et al., 2004, Yeom et al., 2008).

Physical activity levels are determined by individual characteristics and shared factors such as the natural and built environment (McCormack & Shiell, 2011). One key aspect of the natural environment is both presence of, and access to, green spaces which may encourage higher levels of physical activity for recreation and transport (Paquet et al., 2013, Van Cauwenberg et al., 2011). Mobility and function in older adults has been associated with proximity to (Rosso, Auchincloss, & Michael, 2011), and quality of greenspace and green infrastructure in the built environment (Tzoulas et al., 2007), such as the presence of recreational facilities and clean environments (Wu, Prina, & Brayne, 2015), spaces that are designed according to the expressed need of individuals (Ward Thompson, 2013, Kerr et al., 2012), and factors of urban planning and design (Durand, Andalib, Dunton, Wolch, & Pentz, 2011). The relationship between physical activity and greenspace has been shown to be independent of preferences in self-selection of home location (Handy, Cao, & Mokhtarian, 2006). Whilst there is some cross-sectional evidence of a positive association between greenspace, its use for physical activity and health, findings are generally equivocal in the literature. This may in part be due to a lack of prospective studies of physical activity trajectories over time (Lee & Maheswaran, 2011). In addition, few studies have focused on specific domains of physical activity that may be associated with exposure to greenspace (Lachowycz & Jones, 2011). A particular example is recreational walking which makes an important contribution to overall physical activity in older people (Tse, Wong, & Lee, 2015). Finally, the mechanisms and moderators, including personal, social and environmental factors which help to explain the relationship between the environment and physical activity have not been well evaluated (Van Cauwenberg et al., 2011, Annear et al., 2014). For example, dog walkers are more likely to achieve higher levels of physical activity than others (Cutt, Giles-Corti, & Knuiman, 2008), and as dog walking often occurs in greenspace (Richards, McDonough, Edwards, Lyle, & Troped, 2013), it may be one mechanism that explains higher levels of physical activity and sense of community in greener areas (Lachowycz and Jones, 2013, Toohey et al., 2013). This lack of understanding limits our ability to provide greenspace or physical activity interventions that are most supportive of active ageing.

This analysis evaluates the role of greenspace in protecting against decline in physical activity over time in older adults, and considers potential mechanisms. It uses the European Prospective Investigation of Cancer (EPIC)-Norfolk cohort study in the UK, which provides data on a wide range of health and lifestyle factors, obtained over a 7.5 year follow-up period in a population-based sample of more than 25,000 adults (Ward Thompson, 2013).

## 2 Materials and methods

### 2.1 Study design and setting

The initial survey for EPIC-Norfolk was conducted between 1993 and 1997 (First Health Check, 1HC), recruiting 25,639 residents of the region of East Anglia, attending 35 general practice surgeries situated in the county of Norfolk (Day et al., 1999). The sample for this analysis included 15,672 participants with self-reported measures of physical activity from the Second Health Check conducted between 1998 and 2000 (2HC, Follow-up 2, from here referred to as ‘baseline’ for the purposes of this analysis) and a postal questionnaire administered between 2006 and 2009 (from here referred to as ‘follow-up’). This allowed the examination of change in physical activity over time.

### 2.2 Physical activity

Physical activity at baseline and follow-up was self-reported in the validated Physical Activity Questionnaire (EPAQ2) (EPIC-Norfolk, 2016, Wareham et al., 2002). Participants reported the number of times and average duration over the past year which they engaged in different activities, within the domains of recreational, household, transport and occupational activity. Weekly energy expenditure was estimated by multiplying the time spent in each activity (number of hours per week) by the metabolic equivalent cost (MET) of each activity (Ainsworth et al., 2011). Overall physical activity was calculated by summing energy expenditure over all four domains. For this analysis, three measures of physical activity were used: overall and recreational activity plus a third category of activities that we hypothesised might take place outdoors in greenspace – walking, cycling and jogging. Absolute change in each measure of physical activity was calculated by subtracting values at baseline from those at follow-up.

### 2.3 Exposure to neighbourhood greenspace

The main explanatory variable was the percentage of land cover in the participant’s home neighbourhood that was classified as greenspace. This was measured at baseline, unless participants were known to be at a different address by the time of follow-up. In these cases, as information on the exact date of moves was unavailable, we measured the average neighbourhood greenness for the two addresses. The ArcGIS 10.1 geographic information system (GIS) software (ESRI, 2012), was used to delineate neighbourhood boundaries around participants’ home locations defined according to their home postcode (zip code). Every postcode was geo-located using the UK Ordnance Survey Code-Point® database (Ordnance Survey, 2014), which provides a set of coordinates depicting the average latitude and longitude of all mail delivery locations within each postcode. On average, each postcode contains 15 addresses.

Neighbourhoods are typically defined as the area within 800 m (approximating to a ten minute walk) of a home location (Dalton, Jones, Panter, & Ogilvie, 2013). However, recent research from studies employing global positioning systems to track movement suggests that 800m may be overly conservative (Boruff, Nathan, & Nijenstein, 2012), and that individuals typically travel greater distances to access resources and be physically active (Hurvitz & Moudon, 2012). Indeed, Hillsdon, Coombes, Griew, and Jones (2015) suggest that most activity is undertaken outside of the proximal home environment (800 m), even for older adults (56.3%), noting that there was little variation according to age. Given that information on actual movement patterns for the participants of EPIC-Norfolk was not available, the sensitivity of findings to neighbourhood definition was examined by employing three neighbourhood measures: 800 m, 3 km and 5 km. To compute each measure, a circular buffer was used to measure the proportion of the area of each circle that was greenspace.

The estimates of neighbourhood greenspace were generated using data from the Centre for Ecology and Hydrology Land Cover Map of the UK (2007) (Centre for Ecology & Hydrology (CEH), 2013), which is derived from satellite images and digital cartography. It records the dominant land use type, based on a 23 class typology, in 25 m by 25 m size grid cells with greenspace being classified as cells that contain broadleaved and coniferous woodland, arable land, improved grassland, semi-natural grassland, mountain, heath and bog for the purposes of this analysis. All of these types of greenspace are potentially accessible locations for activity participation. In addition to the use of public paths, the ‘right to roam’ in the UK grants people the right to use open access land, which includes common and privately owned land in potentially any of the above land uses. In addition, greenspace in the home neighbourhood may not need to be accessible for it to benefit health, as its presence may inspire individuals to engage in physical activity outside of the home environment. Each participant’s neighbourhood exposure was computed by overlaying the mapped greenspace with the participant’s neighbourhood boundary in the GIS software.

### 2.4 Covariates and confounders

Demographic, lifestyle, health and anthropometric characteristics, collected using the Health and Lifestyle Questionnaire at the initial survey, baseline and follow-up, were chosen for this analysis based on empirical evidence and theoretical relevance of associations with physical activity and greenspace. Covariates included age, sex and BMI at initial survey. The relationship between greenspace and physical activity might be confounded by socio-economic status (SES) (Lee & Maheswaran, 2011), at both the individual and neighbourhood level. Employment derived social class was used at the individual level, obtained at the initial survey, classed as manual (skilled manual, semi-skilled, unskilled) and non-manual (professional, managerial and technical, skilled non-manual). At the neighbourhood level, we used the Townsend Index, a measure of relative deprivation based on information about area employment, car ownership, home ownership and household overcrowding from the UK Census (Townsend, Phillimore, & Beattie, 1988), derived at initial survey. Marital status (Trost, Owen, Bauman, Sallis, & Brown, 2002) at baseline and the presence of mobility limitations (difficulty walking half a mile) at follow-up were also included in the analysis. Dog walking, measured at follow-up, was tested as a potential mediator in the relationship between exposure to greenspace and decline in physical activity (Lachowycz and Jones, 2013, Toohey et al., 2013). Ethnicity has been found to be associated with physical activity (Gill, Celis-Morales, & Ghouri, 2014), but it was not included in this analysis as 99.7% of the sample (N=15,529) were white, reflecting the population of Norfolk, which was 98.5% according to the 2001 Census (Office for National Statistics, 2001).

### 2.5 Data analysis

Baseline characteristics of the sample were compared for participants living in the greenest 25% (quartile) of neighbourhoods versus the least green 25%, using one-way analysis of variance (ANOVA) for continuous variables and chi-square tests for categorical variables. The primary outcome, change in physical activity, followed a normal distribution, therefore parametric tests were used. Change in physical activity between baseline and follow-up according to quartile of greenspace exposure in the home neighbourhood was explored using error bar plots and ANOVA. Multivariable regression models were used to explore the association between exposure to greenspace, divided into quartiles, and change in physical activity between baseline and follow-up. The reference category was individuals living in the least green home neighbourhoods (quartile 1). Models were adjusted for physical activity at baseline, age, sex, BMI, SES and marital status. Mediation analysis was conducted to test dog walking as a potential mediator on the causal path between exposure to greenspace and change in physical activity. While the test cannot prove causation, it can indicate whether the data fit with the presumed causal structure (de Vries, van Dillen, Groenewegen, & Spreeuwenberg, 2013), namely that greenspace facilitates dog walking and thereby physical activity. The product of the coefficients method developed by Preacher and Hayes was followed, to calculate total, direct and indirect effects with bootstrapped, bias corrected, standard errors (Preacher & Hayes, 2008). All analyses were conducted using Stata version 13 (Stata Corp, 2013).

## 3 Results

### 3.1 Sample characteristics

Of the 15,672 participants recruited in the initial survey, we excluded four who did not have a valid postcode that allowed their residential location to be determined. We also excluded a small number of participants (n=32) who had moved far from the study area by the time of the follow-up. A total of 15,636 individuals were included in the analysis with a mean age of 62 years at our baseline. The average length of follow-up was 7.5 years. Our sample therefore represent a cohort that were starting to reach older age in the UK, where state pensionable age was 60 years for women and 65 years for men, at the time of survey (now 63 years for women) (Gov.uk, 2016). There were statistically significant differences (P<0.001) between participant characteristics and quartile of greenspace in terms of age, social class, neighbourhood deprivation, marital status, mobility limitations, dog ownership and walkers, and the urban/rural nature of the home location (Table 1). The larger magnitude of differences were observed for those with professional and managerial occupations, for those who were married, for people owning and walking dogs, for people living in affluent areas, and for people living in urban versus rural locations. Of the total sample, we know that 393 people (2.5%) had moved house by the time of follow-up.

**Table 1:** Baseline characteristics of participants in EPIC Norfolk, according to percentage of greenspace (least green 25% and most green 25%) in their home neighbourhood.

Characteristic	All	Least green 25%	Most green 25%	Difference, least and most green	*P
Age at baseline (years)	62.2±9.1 (15,632)	62.6±9.1 (3909)	61.2±9.0 (3908)	1.4	<0.001
Waist/hip ratio	0.85±0.09 (14,848)	0.85±0.09 (3647)	0.85±0.09 (3726)	0.0	0.558
BMI (kg/m^2^)	26.7±4.0 (15,464)	26.7±4.1 (3878)	26.6±3.9 (3875)	0.1	0.374
Social class (count)					<0.001
Professional	7.4 (1134)	6.8 (260)	8.2 (314)	1.4	
Managerial	38.8 (5951)	34.6 (1322)	46.2 (1170)	11.6	
Skilled non manual	16.7 (2559)	17.3 (660)	13.2 (504)	4.1	
Skilled manual	21.5 (3303)	24.1 (923)	18.5 (710)	5.6	
Semi-skilled	12.6 (1929)	13.3 (507)	11.6 (443)	1.7	
Unskilled	3.1 (479)	3.9 (150)	2.4 (91)	1.5	
Neighbourhood deprivation (count)					<0.001
Relatively affluent	85.1 (13,299)	67.3 (2632)	95.7 (3741)	28.4	
Relatively deprived	14.9 (2337)	32.7 (1279)	4.3 (168)	28.4	
Marital status (count)					<0.001
Single	4.3 (662)	5.9 (231)	3.5 (137)	2.4	
Married	79.9 (12,429)	74.2 (2882)	84.5 (3284)	10.3	
Separated or divorced	6.5 (1009)	9.1 (352)	4.8 (185)	4.3	
Widowed	9.4 (1455)	10.8 (418)	7.2 (31)	3.6	
Mobility: limited walking half a mile (count)					0.001
Yes (a lot)	9.1 (1115)	10.2 (306)	7.9 (248)	2.3	
Yes (limited)	12.5 (1532)	13.3 (397)	11.8 (369)	1.5	
No	78.4 (9617)	76.5 (2285)	80.3 (2517)	3.8	
Dog owners (count)	18.4 (1992)	11.9 (317)	28.7 (780)	16.8	<0.001
Dog walking (count)					<0.001
Not applicable, don’t own a dog	77.9 (8427)	83.3 (2212)	68.2 (1853)	15.0	
Never	4.4 (475)	4.6 (123)	5.0 (137)	0.4	
Sometimes, but not every day	5.6 (606)	3.8 (100)	8.0 (218)	4.3	
Once a day	6.3 (687)	4.9 (130)	9.6 (261)	4.7	
More than once a day	5.8 (628)	3.5 (92)	9.1 (247)	5.6	
Urban/rural location (count)					<0.001
Urban	45.3 (7079)	81.5 (3188)	0.3 (12)	81.2	
Town and fringe	22.3 (3483)	14.5 (567)	2.3 (90)	12.2	
Village	23.3 (3649)	2.5 (98)	69.3 (2707)	66.7	
Hamlet/isolated dwelling	9.1 (1425)	1.5 (58)	28.1 (1100)	26.7	
Greenspace (%)	56.6 (31.4)	15.2 (9.1)	95.7 (3.5)	80.5	

Results are % or mean±SD (n). *P-values from ANOVA and chi square, testing for significant differences between all four quartiles of greenspace and each characteristic.

### 3.2 Unadjusted analysis of change in physical activity

**Fig. 1:**
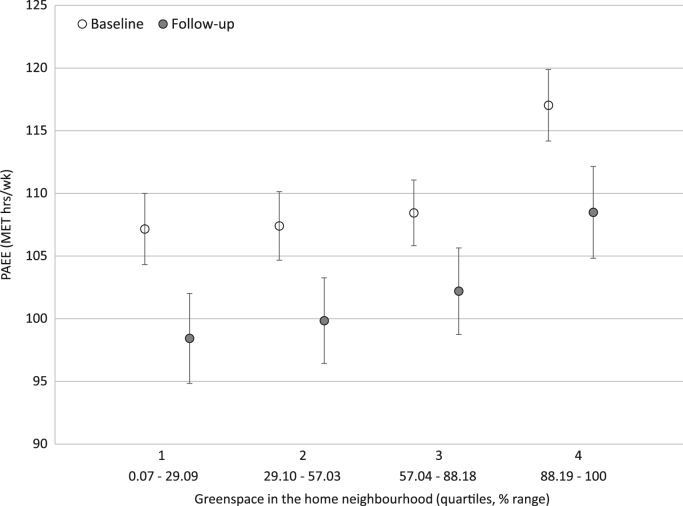
Mean (95% CI) overall physical activity energy expenditure (PAEE) at baseline and follow-up by quartile of greenspace in the home neighbourhood.

**Fig. 2:**
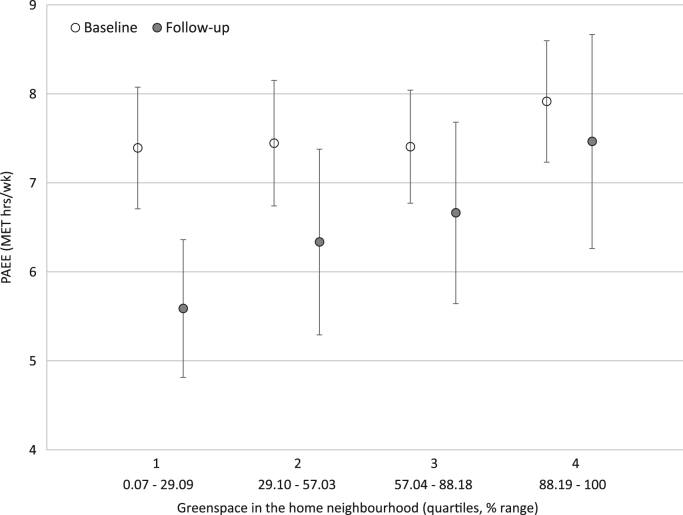
Mean (95% CI) outdoor physical activity energy expenditure (PAEE) at baseline and follow-up by quartile of greenspace in the home neighbourhood.

**Fig. 3:**
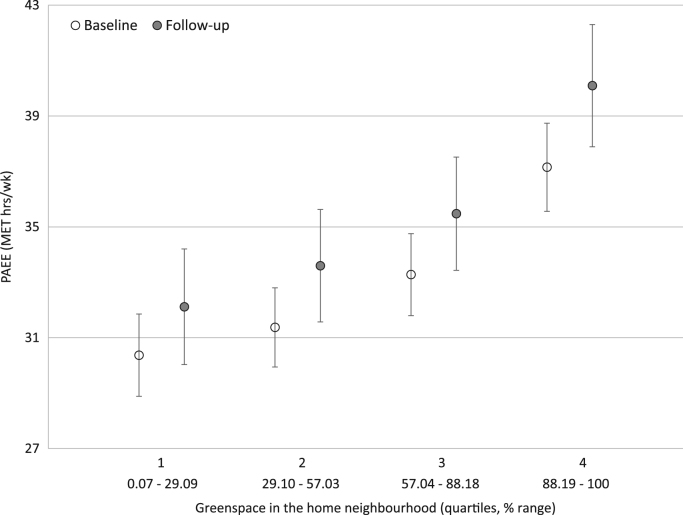
Mean (95% CI) recreational physical activity energy expenditure (PAEE) at baseline and follow-up by quartile of greenspace in the home neighbourhood.

Participants in the greenest quartile of home neighbourhoods were more physically active overall at baseline than those in the least green quartile (mean 117.0 versus 107.2 MET hours per week, P<0.001, Fig. 1). Participants experienced a decline in physical activity between baseline and follow-up of 12.6 MET hours per week (hrs/wk) overall. This decline was significantly different according to quartile of greenspace (P=0.041). Decline was less for those in greener neighbourhoods, as physical activity declined by 12.6 MET h/wk in the most green areas, but 14.2 MET hrs/wk in the least green. For activity in outdoor locations, participants overall experienced an average decline of 1.0 MET h/wk between baseline and follow-up. Again, the decline was less in greener neighbourhoods, with a 0.5 MET h/wk reduction in the most green areas but a larger 1.8 MET h/wk decline in the least green (P=0.042, Fig. 2). Conversely, recreational physical activity increased slightly between baseline and follow-up, and this increase was greatest for those in the greenest neighbourhoods (mean 2.0 MET hours per week) compared to those in the least green (mean 0.5 MET hours per week) (Fig. 3), although this difference was not statistically significant (P=0.377).

### 3.3 Adjusted analysis of change in physical activity

Table 2 presents the results of the regression analysis for change in overall physical activity according to greenspace in the home neighbourhood, adjusted for baseline physical activity. After adjustment for baseline physical activity, participants living in the greenest areas experienced a slower decline in overall physical activity, with a difference of 5.6 MET h/wk compared to those living in the least green (Model 1). Higher baseline physical activity was associated with a greater mean difference in physical activity change. The trend across greenspace quartiles was highly statistically significant with the greenest areas being most protective of decline (P<0.001). When adjusted for additional significant covariates of age, sex, BMI, social class and marital status (Model 2), living in the greenest home neighbourhoods at baseline was protective against decline, with these participants showing a difference in overall physical activity of 4.2 MET h/wk (trend P=0.001) from those in the least green neighbourhoods. The adjusted R-squared values indicated that the fully adjusted model explained just over a quarter (26.5%) of the variance in change in overall physical activity.

**Table 2:** Regression models for change in overall physical activity between baseline and follow-up, according to quartile of greenspace.

	Model 1 Adjusted for baseline PA (n=10997, adjusted R^2^ 19.4%)					Model 2 Adjusted for baseline PA, age, sex, BMI, social class and marital status (n=10785, adjusted R^2^ 26.5%)				
		95% CI					95% CI			
	Coeff.	Lower	Upper	P	P trend	Coeff.	Lower	Upper	P	P trend
Quartile of greenspace: * quartile 1 (least green, ref)*	1.00				<0.001	1.00				0.001
* quartile 2*	1.19	−1.49	3.87	0.384		1.51	−1.07	4.09	0.251	
* quartile 3*	4.01	1.33	6.69	0.003		3.07	0.48	5.66	0.020	
* quartile 4 (most green)*	5.56	2.88	8.24	<0.001		4.21	1.60	6.81	0.002	
Baseline PA (MET h/wk)	−0.46	−0.48	−0.44	<0.001		−0.58	−0.60	−0.56	<0.001	
Age at 2HC (years)						−1.84	−1.96	−1.72	<0.001	
Sex (ref=female)						−3.51	−5.39	−1.64	<0.001	
BMI (kg/m^2^)						−0.48	−0.71	−0.24	<0.001	
Social class (ref=non-manual)						1.16	−0.76	3.07	0.236	
Marital status (ref=not married)						3.96	1.51	6.41	0.002	
*Constant*	*37.75*	*35.00*	*40.50*	<*0.001*		*174.49*	*164.09*	*184.88*	<*0.001*	

Models of change in recreational physical activity (Table 3) followed a similar pattern to overall activity, where participants living in the greenest areas presented a mean difference of 4.9 MET h/wk more than participants living in the least green when adjusted for baseline activity (Model 1), reducing to 4.0 MET h/wk when adjusted for age, sex, BMI, social class and marital status (Model 2). Participants living in the greenest areas also experienced a slower decline in outdoor physical activity (Table 4) with a difference of 1.7 MET h/wk compared to those in the least green areas (Model 1), reducing to 1.3 MET h/wk when adjusted for age, sex, BMI, social class and marital status (Model 2). Neighbourhood deprivation was not statistically significantly associated with outcomes in any of the models.

**Table 3:** Regression models for change in recreational physical activity between baseline and follow-up, according to quartile of greenspace.

	Model 1 Adjusted for baseline PA (n=10852, adjusted R^2^ 19.3%)					Model 2 Adjusted for baseline PA, age, sex, BMI, social class and marital status (n=10649, adjusted R^2^ 21.7%)				
		95% CI					95% CI			
	Coeff.	Lower	Upper	P	P trend	Coeff.	Lower	Upper	P	P trend
Quartile of greenspace: * quartile 1 (least green, ref)*	1.00				<0.001	1.00				<0.001
* quartile 2*	0.92	−0.75	2.59	0.279		0.87	−0.79	2.52	0.306	
* quartile 3*	2.02	0.35	3.69	0.018		1.53	−0.13	3.19	0.071	
* quartile 4 (most green)*	4.89	3.22	6.56	<0.001		4.03	2.36	5.71	<0.001	
Baseline PA (MET h/wk)	−0.52	−0.54	−0.50	<0.001		−0.53	−0.55	−0.51	<0.001	
Age at 2HC (years)						−0.50	−0.57	−0.43	<0.001	
Sex (ref=female)						6.16	4.94	7.39	<0.001	
BMI (kg/m^2^)						−0.28	−0.43	−0.13	<0.001	
Social class (ref=non-manual)						−0.74	−1.97	0.48	0.233	
Marital status (ref=not married)						2.33	0.76	3.91	0.004	
*Constant*	*16.79*	*15.45*	*18.13*	<*0.001*		*51.10*	*45.19*	*57.00*	<*0.001*

**Table 4:** Regression models for change in outdoor physical activity between baseline and follow-up, according to quartile of greenspace.

	Model 1 Adjusted for baseline PA (n=15636, adjusted R^2^ 19.8%)					Model 2 Adjusted for baseline PA, age, sex, BMI, social class and marital status (n=15116, adjusted R^2^ 19.8%)				
		95% CI					95% CI			
	Coeff.	Lower	Upper	P	P trend	Coeff.	Lower	Upper	P	P trend
Quartile of greenspace: * quartile 1 (least green, ref)*	1.00				<0.001	1.00				0.007
* quartile 2*	0.73	−0.14	1.61	0.101		0.77	−0.13	1.67	0.095	
* quartile 3*	1.07	0.20	1.95	0.017		0.85	−0.06	1.75	0.066	
* quartile 4 (most green)*	1.74	0.86	2.62	<0.001		1.28	0.38	2.19	0.006	
Baseline PA (MET h/wk)	−0.74	−0.76	−0.71	<0.001		−0.74	−0.76	−0.71	<0.001	
Age at 2HC (years)						−0.19	−0.22	−0.15	<0.001	
Sex (ref=female)						0.53	−0.12	1.18	0.112	
BMI (kg/m^2^)						−0.16	−0.24	−0.08	<0.001	
Social class (ref=non-manual)						−0.71	−1.37	−0.05	0.036	
Marital status (ref=not married)						1.03	0.20	1.86	0.015	
*Constant*	*3.64*	*3.00*	*4.29*	<*0.001*		*19.11*	*15.93*	*22.30*	<*0.001*	

To put these results into context, in fully adjusted analysis, the model coefficients predict that the average participant experienced a decline of 8.0 MET h/wk for overall activity if they lived in the greenest neighbourhoods against a predicted decline of 12.1 MET h/wk for participants in the least green neighbourhoods. Corresponding values were an increase of 1.7 versus a decline of 0.02 MET h/wk for recreational physical activity, and an increase of 7.3 versus a decline of 0.4 MET h/wk for outdoor physical activity. A value of 3.5 MET h/wk is equivalent to an hour of walking at a moderate pace on a firm, level surface.

Mediation analysis suggested that dog walking partially mediated the association between exposure to greenspace and change in physical activity (Table 5). For overall physical activity, 22.6% of the total effect is mediated, accounting for baseline activity, age, sex, BMI, social class and marital status. The mediated percentage increased to 28.1% for recreational physical activity and 50.0% for outdoor physical activity.

**Table 5:** Total, direct, and indirect effect, via the mediator of dog walking, of exposure to green space on change in physical activity.

		95% CI			
Effect (on PA change) Ref=least green quartile	Coef.	Lower	Upper	St. error	P
*Overall physical activity* (*n*=10573)^a^					
Total effect	8.33	1.90	14.77	3.28	0.011
Direct effect	6.45	0.01	12.89	3.28	0.050
Indirect effect (through dog walking)	1.88	1.25	2.78	0.35	<0.001

*Recreational physical activity* (*n*=10446)^b^					
Total effect	6.20	2.06	10.34	2.11	0.003
Direct effect	4.46	0.33	8.58	2.11	0.034
Indirect effect (through dog walking)	1.74	1.22	2.35	0.27	<0.001

*Outdoor physical activity* (*n*=10616)^c^					
Total effect	4.16	1.08	7.24	1.57	0.008
Direct effect	2.08	-0.97	5.14	0.28	0.182
Indirect effect (through dog walking)	2.08	1.50	2.74	0.28	<0.001

Least green quartile versus all other quartiles of home neighbourhoods. Coefficients with 95% confidence intervals (bias corrected for indirect effects) and significance values (P). All models are adjusted for baseline physical activity, age, sex, BMI, social class and marital status.

a Percent mediated 22.6%.

b Percent mediated 28.1%.

c Percent mediated 50.0%.

Fitting an interaction term to the regression models suggested there were no statistically significant differences according to urban-rural status in associations with the level of greenspace for each domain of physical activity.

Sensitivity analysis (Supplemental File 1) suggested that the size of neighbourhood used to explore exposure to greenspace did not affect the associations with change in physical activity. The effect of dog walking as a mediator remained statistically significant at P<0.001 across all neighbourhood sizes, and the effect size reduced only slightly with increasing neighbourhood size.

## 4 Discussion

### 4.1 Implications

Greener local neighbourhoods appear to be protective against decline in overall, outdoor and recreational physical activity in the EPIC-Norfolk cohort, supporting the findings of previous studies (Paquet et al., 2013, Van Cauwenberg et al., 2011, Rosso et al., 2011). There was a strong association between physical activity change during the mean 7.5 years between baseline and follow-up in the cohort and how green the home neighbourhood was during this period, taking into account physical activity at baseline. Participants living in the greenest home neighbourhoods at baseline experienced a significantly slower decline in physical activity of over 4 MET h/wk for overall and recreational physical activity, and 1.3 MET h/wk for outdoor activity, when compared to those in the least green areas. The relationship did not change substantially after adjustment for covariates of age, sex, BMI, social class and marital status.

Dog walking was found to quite strongly mediate the relationship between exposure to greenspace and physical activity change, particularly for outdoor activity, where 50% of the relationship was via this pathway. These findings support prior evidence that dog walking may be a way to facilitate regular physical activity (Cutt et al., 2008) and social interactions (Knight & Edwards, 2008), particularly in older populations (Toohey et al., 2013). However, the results from this cross sectional analysis cannot provide causality and further investigation is required to establish if the hypothesised mechanism - that greenspace facilitates dog walking and thereby physical activity – is in fact operating. If so, designing interventions to promote and support dog walking, perhaps through education and social support to increase self-efficacy (Richards et al., 2013), may be advantageous. Promoting dog ownership may be a further strategy to deliver social and psychological benefits, although alternatives, such as dog-sharing (e.g. www.BorrowMyDoggy.com), fostering, or companion animal policies such as dog walking programs (Johnson & Meadows, 2010), may need to be available to those who cannot look after an animal all of the time (Knight & Edwards, 2008).

Other causal mechanism(s) behind the observed association(s) between exposure to greenspace and change in physical activity exist. For example, older people may be active in greenspace due to participation in group activities and social interactions (Lachowycz & Jones, 2013), such as walking groups, which have been shown to increase physical activity particularly for adults over 60 years of age (Kassavou, Turner, & French, 2013). Further, where greenspace is not easily accessible, innovative solutions may be necessary, such as mall-walking, which has been shown to improve the health of older people through increasing physical activity (Farren et al., 2015).

### 4.2 Strengths and limitations

The research has a number of strengths. EPIC-Norfolk provided a large sample (n=15,672), with two sets of detailed physical activity measurements an average of 7.5 years apart. The sample was drawn from a variety of urban and rural locations across the county for high exposure heterogeneity. We used information about the specific domains of physical activity likely to be conducted outside, including recreational walking, jogging and cycling. Whilst we had no information about use of greenspace amongst our participants, using these specific domains is progress towards addressing limitations outlined in previous analyses, whereby aggregating domains of activity may obscure relationships between specific types of activity and environmental characteristics (Van Cauwenberg et al., 2011). We used mediation analysis to investigate the possible causal mechanism of dog walking with the observed associations. Home neighbourhood buffers were computed based on the home address of individuals. As it is unclear if greenspace needs to be publicly accessible or just visible to encourage physical activity, circular buffers were used to represent the level of green around the home location rather than zones delineated according to road network distances.

In terms of limitations, we were not able to assess quality of greenspaces within the neighbourhood, despite some research suggesting that more attractive, larger spaces, with certain amenities encourage higher levels of physical activity (Ward Thompson, 2013, Lachowycz and Jones, 2011). In addition, we were not able to identify which greenspace was publicly accessible in the data. Nevertheless, greenspace in the home neighbourhood may not need to be accessible for it to benefit health, as its presence may inspire individuals to engage in physical activity outside of the home environment. In the absence of data on quality or accessibility of greenspace, we used detailed land cover information with circular buffers to objectively indicate a potential maximum accessible greenspace in neighbourhoods. We also tested different classifications of exposure to greenspace by running the models on different neighbourhood buffer sizes, based on evidence that people may roam further than their immediate home neighbourhood (Hillsdon et al., 2015), which did not strongly affect the association between exposure and decline in physical activity. However, it is noteworthy that evidence as to what is an appropriate ‘neighbourhood’ for older adults is unclear, and they may tend to stay closer to home than younger individuals (Jansen et al., 2016, Prins et al., 2014). The nature, meaning and use of greenspace may differ between urban and rural areas, with green urban areas particularly tending to be accessible and managed. However, stratification by urban-rural status revealed no evidence of moderation effects in this dataset, although this may be due to the fact that just 12 of our participants lived in urban neighbourhoods that fell within the top quartile of greenness.

We did not have the exact house location for participants, so we used postcodes to classify exposure. Therefore, potential error in measurement exists due to the difference in location between postcode centroids and the exact address, the magnitude of which may be greater in rural postcode zones which cover larger areas on average (15.6 ha) than urban (1.3 ha). However, even in rural settings errors in the estimates of neighbourhood greenness are likely to be small given that our 800m based neighbourhoods are 201 ha in size. Potential bias may exist in this study, as certain characteristics, such as those owning dogs or those wishing to be more active, may intentionally self-select neighbourhoods with greater availability of neighbourhood greenspace (McCormack & Shiell, 2011). Nevertheless, there is empirical evidence to suggest that any effect of self-selection in studies of the built environment and health may not be large (James et al., 2015) and may in fact tend to bias associations towards the null rather than produce false-positives (Boone-Heinonen, Guilkey, Evenson, & Gordon-Larsen, 2010). As our primary exposure was predominantly measured at a single time point, we are limited in our ability to ascribe causality to the associations detected.

We measured physical activity with self-reported data, which may be subject to error (Prince et al., 2008), and the use of METs introduces a potential source of error, as it is based on an average individual. Nevertheless, the measurement tool and methodology we used has been shown to be both valid and repeatable (The InterAct Consortium, 2012, Wareham et al., 2003). It is possible that our assumed outdoor activities of walking, jogging and cycling, could be undertaken indoors, for example using gym equipment. However, we believe that participants would be more likely to record in a separate category of the questionnaire covering ‘Conditioning exercises e.g. exercise bikes or rowing machines’. One other limitation was that the study was conducted in an English county and may not be representative of other areas. In particular, there was a lack of ethnic heterogeneity in the sample, with as over 99% of the participants stating their ethnicity were white. Additionally, the self-report nature of physical activity is a limitation, although it allowed us look at types of activity that may particularly be undertaken in greenspace, which is not easy to ascertain from accelerometery.

## 5 Conclusions

Greener home neighbourhoods appear to offer protection against decline in physical activity in older people. Dog walking explains half of this association for outdoor physical activity, so our findings suggest it should be actively promoted to facilitate regular physical activity and maintenance of mobility in later life. Future research should explore other potential mechanisms that may elucidate the unexplained components of the association between greenspace and change in physical activity over time.

## Competing interests

The authors declare they have no actual or potential competing interests, financial or otherwise.
