# Supplementary material for: Neighbourhood greenspace is associated with a slower decline in physical activity in older adults: A prospective cohort study
Source: SSM Popul Health. 2016 Sep 14;2:683–91. doi: 10.1016/j.ssmph.2016.09.006 (PMC5165047; doi:10.1016/j.ssmph.2016.09.006)
Supplement: Supplementary file 1 — Supplementary material [file mmc1.docx]

**Supplementary file**

**Sensitivity analysis**

Results of regression and mediation analysis, using alternative neighbourhood buffer sizes of 800m, 3km and 5km

Regression models for change in **overall physical activity** between baseline and follow-up.

| 800m |  | Adjusted for baseline PA, age, sex, BMI, social class and marital status  (n=10785, adjusted R^2^ 26.5%) | | | | |
| --- | --- | --- | --- | --- | --- | --- |
|  |  |  | 95% CI | |  |  |
|  |  | Coeff. | Lower | Upper | P | P trend |
| Quartile of greenspace:  *quartile 1 (least green, ref)* |  | 1.00 |  |  |  | 0.001 |
| *quartile 2* |  | 1.51 | -1.07 | 4.09 | 0.251 |  |
| *quartile 3* |  | 3.07 | 0.48 | 5.66 | 0.020 |  |
| *quartile 4 (most green)* |  | 4.21 | 1.60 | 6.81 | 0.002 |  |
| Baseline PA (MET hrs/wk) |  | -0.58 | -0.60 | -0.56 | <0.001 |  |
| Age at 2HC (years) |  | -1.84 | -1.96 | -1.72 | <0.001 |  |
| Sex (ref=female) |  | -3.51 | -5.39 | -1.64 | <0.001 |  |
| BMI (kg/m^2^) |  | -0.48 | -0.71 | -0.24 | <0.001 |  |
| Social class (ref=non-manual) |  | 1.16 | -0.76 | 3.07 | 0.236 |  |
| Marital status (ref=not married) |  | 3.96 | 1.51 | 6.41 | 0.002 |  |
| *Constant* |  | *174.49* | *164.09* | *184.88* | *<0.001* |  |

| 3km |  | Adjusted for baseline PA, age, sex, BMI, social class and marital status  (n=10649, adjusted R^2^ 26.5%) | | | | |
| --- | --- | --- | --- | --- | --- | --- |
|  |  |  | 95% CI | |  |  |
|  |  | Coeff. | Lower | Upper | P | P trend |
| Quartile of greenspace:  *quartile 1 (least green, ref)* |  | 1.00 |  |  |  | 0.004 |
| *quartile 2* |  | 0.20 | -2.37 | 2.77 | 0.880 |  |
| *quartile 3* |  | 1.81 | -0.79 | 4.41 | 0.171 |  |
| *quartile 4 (most green)* |  | 3.46 | 0.88 | 6.05 | 0.009 |  |
| Baseline PA (MET hrs/wk) |  | -0.58 | -0.60 | -0.56 | <0.001 |  |
| Age at 2HC (years) |  | -1.84 | -1.96 | -1.72 | <0.001 |  |
| Sex (ref=female) |  | -3.50 | -5.37 | -1.62 | <0.001 |  |
| BMI (kg/m^2^) |  | -0.49 | -0.72 | -0.25 | <0.001 |  |
| Social class (ref=non-manual) |  | 1.02 | -0.89 | 2.93 | 0.296 |  |
| Marital status (ref=not married) |  | 4.07 | 1.61 | 6.52 | 0.001 |  |
| *Constant* |  | *175.60* | *165.24* | *185.96* | *<0.001* |  |

| 5km |  | Adjusted for baseline PA, age, sex, BMI, social class and marital status  (n=10986, adjusted R^2^ 26.5%) | | | | |
| --- | --- | --- | --- | --- | --- | --- |
|  |  |  | 95% CI | |  |  |
|  |  | Coeff. | Lower | Upper | P | P trend |
| Quartile of greenspace:  *quartile 1 (least green, ref)* |  | 1.00 |  |  |  | 0.008 |
| *quartile 2* |  | 0.77 | -1.78 | 3.33 | 0.553 |  |
| *quartile 3* |  | 1.29 | -1.30 | 3.89 | 0.329 |  |
| *quartile 4 (most green)* |  | 3.53 | 0.95 | 6.12 | 0.007 |  |
| Baseline PA (MET hrs/wk) |  | -0.58 | -0.60 | -0.56 | <0.001 |  |
| Age at 2HC (years) |  | -1.85 | -1.96 | -1.73 | <0.001 |  |
| Sex (ref=female) |  | -3.48 | -5.35 | -1.60 | <0.001 |  |
| BMI (kg/m^2^) |  | -0.49 | -0.72 | -0.25 | <0.001 |  |
| Social class (ref=non-manual) |  | 0.94 | -0.97 | 2.86 | 0.335 |  |
| Marital status (ref=not married) |  | 4.11 | 1.66 | 6.56 | 0.001 |  |
| *Constant* |  | *175.80* | *165.40* | *186.19* | *<0.001* |  |

Regression models for change in **recreational physical activity** between baseline and follow-up.

| 800m |  | Adjusted for baseline PA, age, sex, BMI, social class and marital status  (n=10649, adjusted R^2^ 21.7%) | | | | |
| --- | --- | --- | --- | --- | --- | --- |
|  |  |  | 95% CI | |  |  |
|  |  | Coeff. | Lower | Upper | P | P trend |
| Quartile of greenspace:  *quartile 1 (least green, ref)* |  | 1.00 |  |  |  | <0.001 |
| *quartile 2* |  | 0.87 | -0.79 | 2.52 | 0.306 |  |
| *quartile 3* |  | 1.53 | -0.13 | 3.19 | 0.071 |  |
| *quartile 4 (most green)* |  | 4.03 | 2.36 | 5.71 | <0.001 |  |
| Baseline PA (MET hrs/wk) |  | -0.53 | -0.55 | -0.51 | <0.001 |  |
| Age at 2HC (years) |  | -0.50 | -0.57 | -0.43 | <0.001 |  |
| Sex (ref=female) |  | 6.16 | 4.94 | 7.39 | <0.001 |  |
| BMI (kg/m^2^) |  | -0.28 | -0.43 | -0.13 | <0.001 |  |
| Social class (ref=non-manual) |  | -0.74 | -1.97 | 0.48 | 0.233 |  |
| Marital status (ref=not married) |  | 2.33 | 0.76 | 3.91 | 0.004 |  |
| *Constant* |  | *51.10* | *45.19* | *57.00* | *<0.001* |  |

| 3km |  | Adjusted for baseline PA, age, sex, BMI, social class and marital status  (n=10649, adjusted R^2^ 21.6%) | | | | |
| --- | --- | --- | --- | --- | --- | --- |
|  |  |  | 95% CI | |  |  |
|  |  | Coeff. | Lower | Upper | P | P trend |
| Quartile of greenspace:  *quartile 1 (least green, ref)* |  | 1.00 |  |  |  | 0.001 |
| *quartile 2* |  | 0.67 | -0.98 | 2.32 | 0.425 |  |
| *quartile 3* |  | 0.39 | -1.28 | 2.06 | 0.646 |  |
| *quartile 4 (most green)* |  | 3.12 | 1.46 | 4.79 | <0.001 |  |
| Baseline PA (MET hrs/wk) |  | -0.52 | -0.54 | -0.50 | <0.001 |  |
| Age at 2HC (years) |  | -0.51 | -0.58 | -0.44 | <0.001 |  |
| Sex (ref=female) |  | 6.17 | 4.94 | 7.40 | <0.001 |  |
| BMI (kg/m^2^) |  | -0.28 | -0.43 | -0.13 | <0.001 |  |
| Social class (ref=non-manual) |  | -0.88 | -2.10 | 0.34 | 0.159 |  |
| Marital status (ref=not married) |  | 2.45 | 0.87 | 4.03 | 0.002 |  |
| *Constant* |  | *51.97* | *46.09* | *57.84* | *<0.001* |  |

| 5km |  | Adjusted for baseline PA, age, sex, BMI, social class and marital status  (n=10649, adjusted R^2^ 21.6%) | | | | |
| --- | --- | --- | --- | --- | --- | --- |
|  |  |  | 95% CI | |  |  |
|  |  | Coeff. | Lower | Upper | P | P trend |
| Quartile of greenspace:  *quartile 1 (least green, ref)* |  | 1.00 |  |  |  | 0.004 |
| *quartile 2* |  | 1.26 | -0.39 | 2.90 | 0.134 |  |
| *quartile 3* |  | 0.89 | -0.78 | 2.56 | 0.296 |  |
| *quartile 4 (most green)* |  | 2.74 | 1.07 | 4.40 | 0.001 |  |
| Baseline PA (MET hrs/wk) |  | -0.52 | -0.54 | -0.50 | <0.001 |  |
| Age at 2HC (years) |  | -0.51 | -0.58 | -0.44 | <0.001 |  |
| Sex (ref=female) |  | 6.15 | 4.92 | 7.38 | <0.001 |  |
| BMI (kg/m^2^) |  | -0.28 | -0.43 | -0.13 | <0.001 |  |
| Social class (ref=non-manual) |  | -0.90 | -2.12 | 0.33 | 0.151 |  |
| Marital status (ref=not married) |  | 2.44 | 0.87 | 4.02 | 0.002 |  |
| *Constant* |  | *51.96* | *46.06* | *57.85* | *<0.001* |  |

Regression models for change in **outdoor physical activity** between baseline and follow-up.

| 800m |  | Adjusted for baseline PA, age, sex, BMI, social class and marital status  (n=15116, adjusted R^2^ 19.8%) | | | | |
| --- | --- | --- | --- | --- | --- | --- |
|  |  |  | 95% CI | |  |  |
|  |  | Coeff. | Lower | Upper | P | P trend |
| Quartile of greenspace:  *quartile 1 (least green, ref)* |  | 1.00 |  |  |  | 0.007 |
| *quartile 2* |  | 0.77 | -0.13 | 1.67 | 0.095 |  |
| *quartile 3* |  | 0.85 | -0.06 | 1.75 | 0.066 |  |
| *quartile 4 (most green)* |  | 1.28 | 0.38 | 2.19 | 0.006 |  |
| Baseline PA (MET hrs/wk) |  | -0.74 | -0.76 | -0.71 | <0.001 |  |
| Age at 2HC (years) |  | -0.19 | -0.22 | -0.15 | <0.001 |  |
| Sex (ref=female) |  | 0.53 | -0.12 | 1.18 | 0.112 |  |
| BMI (kg/m^2^) |  | -0.16 | -0.24 | -0.08 | <0.001 |  |
| Social class (ref=non-manual) |  | -0.71 | -1.37 | -0.05 | 0.036 |  |
| Marital status (ref=not married) |  | 1.03 | 0.20 | 1.86 | 0.015 |  |
| *Constant* |  | *19.11* | *15.93* | *22.30* | *<0.001* |  |

| 3km |  | Adjusted for baseline PA, age, sex, BMI, social class and marital status  (n=15116, adjusted R^2^ 19.8%) | | | | |
| --- | --- | --- | --- | --- | --- | --- |
|  |  |  | 95% CI | |  |  |
|  |  | Coeff. | Lower | Upper | P | P trend |
| Quartile of greenspace:  *quartile 1 (least green, ref)* |  | 1.00 |  |  |  | 0.003 |
| *quartile 2* |  | 0.09 | -0.81 | 0.99 | 0.847 |  |
| *quartile 3* |  | 0.75 | -0.15 | 1.66 | 0.103 |  |
| *quartile 4 (most green)* |  | 1.21 | 0.30 | 2.11 | 0.009 |  |
| Baseline PA (MET hrs/wk) |  | -0.74 | -0.76 | -0.71 | <0.001 |  |
| Age at 2HC (years) |  | -0.19 | -0.22 | -0.15 | <0.001 |  |
| Sex (ref=female) |  | 0.53 | -0.12 | 1.19 | 0.112 |  |
| BMI (kg/m^2^) |  | -0.16 | -0.24 | -0.08 | <0.001 |  |
| Social class (ref=non-manual) |  | -0.73 | -1.39 | -0.07 | 0.030 |  |
| Marital status (ref=not married) |  | 1.04 | 0.22 | 1.87 | 0.013 |  |
| *Constant* |  | *19.38* | *16.21* | *22.55* | *<0.001* |  |

| 5km |  | Adjusted for baseline PA, age, sex, BMI, social class and marital status  (n=15116, adjusted R^2^ 19.8%) | | | | |
| --- | --- | --- | --- | --- | --- | --- |
|  |  |  | 95% CI | |  |  |
|  |  | Coeff. | Lower | Upper | P | P trend |
| Quartile of greenspace:  *quartile 1 (least green, ref)* |  | 1.00 |  |  |  | 0.007 |
| *quartile 2* |  | 0.48 | -0.42 | 1.38 | 0.295 |  |
| *quartile 3* |  | 0.18 | -0.73 | 1.08 | 0.703 |  |
| *quartile 4 (most green)* |  | 1.42 | 0.51 | 2.32 | 0.002 |  |
| Baseline PA (MET hrs/wk) |  | -0.74 | -0.76 | -0.71 | <0.001 |  |
| Age at 2HC (years) |  | -0.19 | -0.23 | -0.15 | <0.001 |  |
| Sex (ref=female) |  | 0.54 | -0.11 | 1.19 | 0.106 |  |
| BMI (kg/m^2^) |  | -0.16 | -0.24 | -0.08 | <0.001 |  |
| Social class (ref=non-manual) |  | -0.75 | -1.41 | -0.09 | 0.027 |  |
| Marital status (ref=not married) |  | 1.06 | 0.23 | 1.88 | 0.012 |  |
| *Constant* |  | *19.46* | *16.28* | *22.64* | *<0.001* |  |

Total, direct, and indirect effect, via the mediator of dog walking, of exposure to green space on change in physical activity

| 800m |  |  | |  |  |
| --- | --- | --- | --- | --- | --- |
|  |  | 95% CI | |  |  |
| Effect (on PA change) Ref = least green quartile | Coef. | Lower | Upper | St. error | P |
|  |  |  |  |  |  |
| *Overall physical activity (n=10573) ^a^* | | | | | |
| Total effect | 8.33 | 1.90 | 14.77 | 3.28 | 0.011 |
| Direct effect | 6.45 | 0.01 | 12.89 | 3.28 | 0.050 |
| Indirect effect (through dog walking) | 1.88 | 1.25 | 2.78 | 0.35 | <0.001 |
|  |  |  |  |  |  |
| *Recreational physical activity (n=10446) ^b^* | | | | | |
| Total effect | 6.20 | 2.06 | 10.34 | 2.11 | 0.003 |
| Direct effect | 4.46 | 0.33 | 8.58 | 2.11 | 0.034 |
| Indirect effect (through dog walking) | 1.74 | 1.22 | 2.35 | 0.27 | <0.001 |
|  |  |  |  |  |  |
| *Outdoor physical activity (n=10616) ^c^* | | | | | |
| Total effect | 4.16 | 1.08 | 7.24 | 1.57 | 0.008 |
| Direct effect | 2.08 | -0.97 | 5.14 | 0.28 | 0.182 |
| Indirect effect (through dog walking) | 2.08 | 1.50 | 2.74 | 0.28 | <0.001 |

Least green quartile versus all other quartiles of home neighbourhoods. Coefficients with 95% confidence intervals (bias corrected for indirect effects) and significance values (P). All models are adjusted for baseline physical activity, age, sex, BMI, social class and marital status. *^a^ Percent mediated 22.6%; ^b^ Percent mediated 28.1%; ^c^ Percent mediated 50.0%.*

| 3km |  |  | |  |  |
| --- | --- | --- | --- | --- | --- |
|  |  | 95% CI | |  |  |
| Effect (on PA change) Ref = least green quartile | Coef. | Lower | Upper | St. error | P |
|  |  |  |  |  |  |
| *Overall physical activity (n=10573) ^a^* | | | | | |
| Total effect | 5.54 | -0.87 | 11.95 | 3.27 | 0.090 |
| Direct effect | 3.86 | -2.54 | 10.27 | 3.27 | 0.238 |
| Indirect effect (through dog walking) | 1.68 | 1.08 | 2.46 | 0.33 | <0.001 |
|  |  |  |  |  |  |
| *Recreational physical activity (n=10446) ^b^* | | | | | |
| Total effect | 4.39 | 0.27 | 8.51 | 2.10 | 0.037 |
| Direct effect | 2.81 | -1.29 | 6.92 | 2.09 | 0.179 |
| Indirect effect (through dog walking) | 1.58 | 1.09 | 2.14 | 0.27 | <0.001 |
|  |  |  |  |  |  |
| *Outdoor physical activity (n=10616) ^c^* | | | | | |
| Total effect | 3.09 | 0.02 | 6.16 | 1.57 | 0.049 |
| Direct effect | 1.25 | -1.78 | 4.29 | 1.55 | 0.419 |
| Indirect effect (through dog walking) | 1.83 | 1.28 | 2.43 | 0.27 | <0.001 |

Least green quartile versus all other quartiles of home neighbourhoods. Coefficients with 95% confidence intervals (bias corrected for indirect effects) and significance values (P). All models are adjusted for baseline physical activity, age, sex, BMI, social class and marital status. *^a^ Percent mediated 30.3%; ^b^ Percent mediated 35.9%; ^c^ Percent mediated 59.4%.*

| 5km |  |  | |  |  |
| --- | --- | --- | --- | --- | --- |
|  |  | 95% CI | |  |  |
| Effect (on PA change) Ref = least green quartile | Coef. | Lower | Upper | St. error | P |
|  |  |  |  |  |  |
| *Overall physical activity (n=10573) ^a^* | | | | | |
| Total effect | 5.50 | -0.88 | 11.89 | 3.26 | 0.091 |
| Direct effect | 3.98 | -2.40 | 10.36 | 3.26 | 0.222 |
| Indirect effect (through dog walking) | 1.52 | 0.98 | 2.22 | 0.31 | <0.001 |
|  |  |  |  |  |  |
| *Recreational physical activity (n=10446) ^b^* | | | | | |
| Total effect | 5.05 | 0.94 | 9.17 | 2.10 | 0.016 |
| Direct effect | 3.64 | -0.45 | 7.73 | 2.09 | 0.081 |
| Indirect effect (through dog walking) | 1.41 | 0.95 | 1.97 | 0.26 | <0.001 |
|  |  |  |  |  |  |
| *Outdoor physical activity (n=10616) ^c^* | | | | | |
| Total effect | 3.36 | 0.30 | 6.42 | 1.56 | 0.031 |
| Direct effect | 1.71 | -1.31 | 4.74 | 1.54 | 0.267 |
| Indirect effect (through dog walking) | 1.65 | 1.13 | 2.17 | 0.26 | <0.001 |

Least green quartile versus all other quartiles of home neighbourhoods. Coefficients with 95% confidence intervals (bias corrected for indirect effects) and significance values (P). All models are adjusted for baseline physical activity, age, sex, BMI, social class and marital status. *^a^ Percent mediated 27.7%; ^b^ Percent mediated 28.0%; ^c^ Percent mediated 49.0%.*
